# 2023 Japan Society of clinical oncology clinical practice guidelines update for antiemesis

**DOI:** 10.1007/s10147-024-02535-x

**Published:** 2024-05-16

**Authors:** Hirotoshi Iihara, Masakazu Abe, Makoto Wada, Keiko Iino, Tatsuo Akechi, Chiyo K. Imamura, Ayako Okuyama, Keiko Ozawa, Yong-Il Kim, Hidenori Sasaki, Eriko Satomi, Masayuki Takeda, Ryuhei Tanaka, Takako Eguchi Nakajima, Naoki Nakamura, Junichi Nishimura, Mayumi Noda, Kazumi Hayashi, Takahiro Higashi, Narikazu Boku, Koji Matsumoto, Yoko Matsumoto, Kenji Okita, Nobuyuki Yamamoto, Kenjiro Aogi

**Affiliations:** 1 Department of Pharmacy, Gifu University Hospital, 1-1 Yanagido, Gifu, 501-1194 Japan; 2 Department of Obstetrics and Gynecology, Hamamatsu University School of Medicine, 1-20-1 Handayama, Higashi-Ku, Hamamatsu, Shizuoka 431-3192 Japan; 3 Department of Psycho‑Oncology and Palliative Medicine, Osaka International Cancer Institute, 3-1-69, Otemae, Chuo-Ku, Osaka, Osaka 541-8567 Japan; 4 School of Nursing, National College of Nursing, Japan, 1-2-1, Umezono, Kiyose, Tokyo 204-8575 Japan; 5 Department of Psychiatry and Cognitive-Behavioral Medicine, Nagoya City University Graduate School of Medical Sciences, 1 Kawasumi, Mizuho-Cho, Mizuho-Ku, Nagoya, 467-8601 Japan; 6 Advanced Cancer Translational Research Institute, Showa University, 1-5-8 Hatanodai, Shinagawa-Ku, Tokyo, 142-8555 Japan; 7 Graduate School of Nursing Science, St. Luke’s International University, 10-1 Akashi-Cho, Chuo-Ku, Tokyo, 104-0044 Japan; 8 Division of Survivorship, Institute for Cancer Control, National Cancer Center, 5-1-1 Tsukiji, Chuo-Ku, Tokyo, 104-0045 Japan; 9 Division of Medical Oncology, Yodogawa Christian Hospital, 1-7-50 Kunijima, Higasiyodogawa-Ku, Osaka, Osaka 533-0024 Japan; 10 Division of Medical Oncology, Hematology and Infectious Disease, Fukuoka University Hospital, 7-45-1, Nanakuma, Jonan-Ku, Fukuoka, 814-0180 Japan; 11 Department of Palliative Medicine, National Cancer Center Hospital, 5-1-1, Tsukiji, Chuo-Ku, Tokyo, 104-0045 Japan; 12 Department of Cancer Genomics and Medical Oncology, Nara Medical University, 840 Shijo-Cho, Kashihara, Nara 634-8521 Japan; 13 Department of Pediatric Hematology/Oncology, International Medical Center, Saitama Medical University, 1398-1 Yamane, Hidaka, Saitama 350-1298 Japan; 14 Department of Early Clinical Development, Kyoto University Graduate School of Medicine, 54 Kawahara-Cho, Shogoin, Sakyo-ku, Kyoto, 606-8507 Japan; 15 Department of Radiation Oncology, St. Marianna University, 2-16-1, Sugao, Miyamae, Kawasaki, Kanagawa 216-8511 Japan; 16 Department of Gastroenterological Surgery, Osaka International Cancer Institute, 3-1-69, Otemae, Chuo-Ku, Osaka, 541-8567 Japan; 17 Non-Profit Organizaition Sasaeau-Kai “Alpha”, 518-7 Kawado-Cho, Chuo-Ku, Chiba, 260-0802 Japan; 18 Department of Clinical Oncology and Hematology, The Jikei University School of Medicine, 3-25-8 Nishi-Shinnbashi Minatoku, Tokyo, 105-8461 Japan; 19 Department of Public Health and Health Policy, The University of Tokyo, 7-3-1 Hongo, Bunkyo-Ku, Tokyo, 113-0033 Japan; 20 Department of Oncology and General Medicine, IMSUT Hospital, Institute of Medical Science, University of Tokyo, 4-6-1 Shiroganedai, Minato-Ku, Tokyo, 108-8639 Japan; 21 Division of Medical Oncology, Hyogo Cancer Center, 13-70 Kitaoji-Cho, Akashi, Hyogo 673-0021 Japan; 22 Non-Profit Organization Ehime Cancer Support “Orange-No-Kai”, 3-8-24 Furukawaminami, Matsuyama, Ehime 790-0943 Japan; 23 Department of Surgery, Otaru Ekisaikai Hospital, 1-4-1, Inaho, Otaru, Hokkaido 047-0032 Japan; 24 Internal Medicine III, Wakayama Medical University, 811-1 Kimiidera, Wakayama, 641-8509 Japan; 25 Department of Breast Surgery, National Hospital Organization Shikoku Cancer Center, 160 Kou, Minamiumemoto-Machi, Matsuyama, Ehime 791-0280 Japan

**Keywords:** Antiemesis, Nausea, Vomiting, CINV, Cancer chemotherapy, Clinical practice guidelines

## Abstract

**Background:**

The Japan Society of Clinical Oncology Clinical Practice Guidelines for Antiemesis 2023 was extensively revised to reflect the latest advances in antineoplastic agents, antiemetics, and antineoplastic regimens. This update provides new evidence on the efficacy of antiemetic regimens.

**Methods:**

Guided by the Minds Clinical Practice Guideline Development Manual of 2017, a rigorous approach was used to update the guidelines; a thorough literature search was conducted from January 1, 1990, to December 31, 2020.

**Results:**

Comprehensive process resulted in the creation of 13 background questions (BQs), 12 clinical questions (CQs), and three future research questions (FQs). Moreover, the emetic risk classification was also updated.

**Conclusions:**

The primary goal of the present guidelines is to provide comprehensive information and facilitate informed decision-making, regarding antiemetic therapy, for both patients and healthcare providers.

## Introduction

The Japan Society of Clinical Oncology Guidelines for Antiemetic Therapy was developed to appropriately evaluate and manage chemotherapy-induced nausea and vomiting and improve treatment efficacy, thereby improving patient quality of life (QOL) and, ultimately, patient prognosis. The initial Japan Society of Clinical Oncology (JSCO) Clinical Practice Guidelines for Antiemesis were published in 2010, with updates in 2015 (revised edition, version 2) and 2018 (revised edition, version 2.2) [1, 2]. Revisions were made in the third edition to reflect new evidence on antineoplastic agents, antiemetics, antineoplastic regimens. In addition, by appropriately assessing the balance between the benefits and harms of different antiemetic therapies based on the evidence, these guidelines aim to facilitate informed decision-making regarding antiemetic therapy for both patients and healthcare providers.

## Methods

### Guiding principles for the development

The antiemetic guideline update committee consisted of 23 working group members and 18 systematic review team members who are multidisciplinary healthcare professionals with expertise in antiemetic research (physicians, nurses, pharmacists, and epidemiologists), two patient advocates, and two secretaries.

In developing and revising these guidelines, the basic approach is to follow the 'Minds Clinical Practice Guideline Development Manual 2017’ [3]. Questions are developed based on the key clinical issues Table 1. Systematic reviews of each question are conducted, and recommendations are determined based on the obtained results [4]. The quality of evidence and definitions are presented in Table 2. The recommendations are generally presented based on a combination of the direction of the recommendation (two directions) and the strength of the recommendation (two levels), as listed in Table 3. The strengths of the recommendation, quality of evidence, and agreement rate were then concurrently stated. The consensus-building method involves web voting using the GRADE grid. Consensus is achieved when the concentration of votes for a specific statement pertaining to each item exceeds 80%, thereby influencing the determination of recommendations [5]. The working group members and patient advocates conducted the voting. If a consensus was not reached in the first round of voting, discussions were held, and a second round of voting was conducted. If consensus was not reached in the second round, the process and summary of the results were documented in the statement.

**Table 1:** Key clinical issues

1. Provide appropriate antiemetic therapy recommendations based on emetogenic risk and options presented
2. Provide appropriate recommendations for emetogenic risk and antiemetic therapy for novel anticancer therapies (new anticancer drugs and regimens)
3. Provide recommendations for proper evaluation of antiemetic therapy efficacy, prediction of efficacy, and highlight potential side effects
4. Review the health economic evaluation of antiemetic therapy
5. Evaluate the effectiveness of non-pharmacological interventions in antiemetic therapy
6. Consider support systems for proper implementation of antiemetic therapy

**Table 2:** Quality of the evidence and definitions

A (High quality)	Further research is very unlikely to change our confidence in the estimate of effect
B (Moderate quality)	Further research is likely to have an important impact on our confidence in the estimate of effect and may change the estimate
C (Low quality)	Further research is very likely to have an important impact on our confidence in the estimate of effect and is likely to change the estimate
D (Very low quality)	Any estimate of effect is very uncertain

**Table 3:** Strength of the recommendations

		Strength of recommendation	
		Strong	Weak
Direction of recommendations	For	We recommend…	We suggest…
	Against	We recommend not…	We suggest not…

### The classification and terminology of the questions in this guideline

The questions in this guideline comprise “background questions (BQ),” “clinical questions (CQ),” and “future research questions (FQ).” BQs represent fundamental knowledge that includes clinical characteristics, epidemiological features, and the overall flow of medical practice. The BQs encompass widely understood issues requiring documentation in the guidelines. CQs focus on the significant but less familiar issues based on the recent evidences wherein a systematic review has been performed, and evidence-based recommendations can be provided. FQs are unresolved issues wherein a systematic review could not be completed owing to insufficient evidence or other constraints, precluding the formulation of evidence-based recommendations.

### Emetic risk classification of the antineoplastic agents

The classification of emetogenicity for antineoplastic agents is primarily based on the incidence of emesis occurring within 24 h following the administration of antineoplastic agents without prophylactic antiemetic administration. High emetic risk (> 90% patients experience acute emesis), moderate emetic risk (30 < –90% of patients experience acute emesis), low emetic risk (10–30% of patients experience acute emesis), and minimal emetic risk (< 10% of patients experience acute emesis) are determined through non-systematic reviews of randomized controlled trials, analysis of product labeling, the evaluation of emetic classification in other international guidelines, and informal consensus.

### Literature search

For this update, we conducted a literature search covering the period from January 1, 1990, to December 31, 2020, using PubMed, Cochrane Library, and Igaku Chuo Zasshi (ICHUSHI) databases. For questions related to nonpharmacological therapy and patient support, an additional search was conducted using CINAHL. The formula used to search the literature is published on the JSCO website [4].

The priority for literature adoption was as follows: (1) randomized controlled trials, (2) non-randomized comparative trials, (3) single-arm trials, (4) case–control studies, and (5) observational studies that allow the extraction of data for both the group receiving the antiemetic therapy under investigation and the group not receiving it. Case reports and case series studies of poor quality were excluded.

### Conflicts of interest for the guideline

In accordance with the JSCO guidelines for the management of conflicts of interest (COIs), the members of the guideline update working group and those of the systematic review team submitted self-disclosures of financial COIs. The COI Committee reviewed these submissions and confirmed that none of the members had any significant financial COI (http://www.jsco-cpg.jp/).

If a voting member had a COI, such as being a lead author or a corresponding author of a paper related to the evidence that forms the basis of the recommendation, or if they had a financial COI with the company or companies involved in the manufacture or sale of the related drugs or medical devices beyond the criteria outlined in the Japan Medical Association’s “Guidance on Eligibility Criteria for Participation in Clinical Practice Guideline Development,” they abstained from voting.

## Results

### Emetic risk classification of antineoplastic agents

The emetic risks of intravenous and oral antineoplastic agents are shown in Tables 4 and 5, respectively. In addition, the emetogenic properties of certain combination chemotherapies are depicted.

**Table 4:** Emetic risk category for intravenous antineoplastic agents

JSCO emetic risk category	Agent (Regimen)
High emetic risk (emetic frequency: 90% <)	Anthracycline and Cyclophosphamide-based regimens
	FOLFIRINOX in patients with pancreatic cancer
	FOLFOXIRI in patients with colorectal cancer
	Cisplatin
	Cyclophosphamide (1,500 mg/m^2^ ≤)
	Dacarbazine
	Doxorubicin (60 mg/m^2^ ≤)
	Epirubicin (90 mg/m^2^ ≤)
	Ifosfamide (2,000 mg/m^2^/single-dose ≤)
	Melphalan (140 mg/m^2^ ≤)
	Streptozocin
	*Carmustine* (250 mg/m^2^ <)
	*Mechlorethamine*
Moderate emetic risk (emetic frequency: 30% < –90%)	Docetaxel + gemcitabine in patients with carcinoma of unknown primary
	Gemcitabine + cisplatin (25 mg/m^2^) in patients with biliary tract cancer
	Gemcitabine + cisplatin (25 mg/m^2^) + S-1 in patients with biliary tract cancer
	Gemcitabine + nab-paclitaxel in patients with pancreatic cancer
	Gemcitabine + S-1 in patients with pancreatic cancer
	Actinomycin D
	Alemtuzumab
	Amrubicin
	Arsenic trioxide
	Azacitidine
	Bendamustine
	Busulfan
	Carboplatin^*^
	Clofarabine
	Cyclophosphamide (< 1,500 mg/m^2^)
	Cytarabine (1,000 mg/m^2^ <)
	Daunorubicin
	Dinutuximab
	Doxorubicin (< 60 mg/m^2^)
	Enocitabine
	Epirubicin (< 90 mg/m^2^)
	Idarubicin
	Ifosfamide (< 2,000 mg/m^2^/single-dose)
	Inotuzumab ozogamicin
	Irinotecan
	Irinotecan (liposomal)
	Melphalan (< 140 mg/m^2^)
	Methotrexate (250 mg/m^2^ ≤)
	Miriplatin
	Nedaplatin
	Oxaliplatin
	Pirarubicin
	Romidepsin
	Temozolomide
	Thiotepa
	Trabectedin
	Trastuzumab deruxtecan^**^
	*Aldesleukin* (12—15 million IU/m^2^ <)
	*Amifostine* (300 mg/m^2^ <)
	*Carmustine* (≤ 250 mg/m^2^)
	*Daunorubicin and cytarabine liposome*
	*Lurbinectedin*
	*Naxitamab*
	Sacituzumab govitecan^**^
Low emetic risk (emetic frequency: 10–30%)	Atezolizumab
	Axicabtagene ciloleucel
	Blinatumomab
	Bortezomib
	Brentuximab vedotin
	Cabazitaxel
	Carfilzomib
	Cytarabine (≤ 1,000 mg/m^2^)
	Docetaxel
	Doxorubicin (liposomal)
	Elotuzumab
	Enfortumab vedotin
	Eribulin
	Etoposide
	Fluorouracil
	Gemcitabine
	Gemtuzumab ozogamic
	Idecabtagene vicleucel
	Isatuximab
	Lisocabtagen maraleucel
	Methotrexate (50 mg/m^2^ < – < 250 mg/m^2^)
	Mitomycin C
	Mitoxantrone
	Mogamulizumab
	Nab-paclitaxel
	Necitumumab
	Nelarabine
	Nimustine
	Nogitecan
	Paclitaxel
	Pemetrexed
	Pentostatin
	Ranimustine
	Temsirolimus
	Tisagenlecleucel
	Trastuzumab emtansine
	*Aldesleukin* (≤ 12 million IU/m^2^)
	*Amifostine* (≤ 300 mg/m^2^)
	*Amivantamab*
	*Belinostat*
	*Brexucabtagene autoleucel*
	*Catumaxomab*
	*Ciltacabtagene autoleucel*
	*Copanlisib*
	*Decitabine*
	*Floxuridine*
	*Ixabepilone*
	*Loncastuximab tesirine*
	*Mitomycin pyelocalyceal*
	*Moxetumomab pasudotox*
	*Omacetaxine*
	*Tafasitamab*
	*Talimogene laherparepvec*
	*Tisotumab vedotin*
	*Vinflunine*
Minimal emetic risk (emetic frequency: < 10%)	L-asparaginase
	Aflibercept beta
	Avelumab
	Bevacizumab
	Bleomycin
	Cemiplimab
	Cetuximab
	Cetuximab sarotalocan
	Cladribine
	Daratumumab
	Daratumumab・vorhyaluronidase alfa
	Darinaparsin
	Denileukin diftitox
	Durvalumab
	Fludarabine
	Ipilimumab
	Methotrexate (≤ 50 mg/m^2^)
	Nivolumab
	Obinutuzumab
	Panitumumab
	Pembrolizumab
	Peplomycin
	Pertuzumab
	Polatuzumab vedotin
	Pralatrexate
	Ramucirumab
	Rituximab
	Talaporfin
	Trastuzumab
	Tremelimumab
	Vinblastine
	Vincristine
	Vindesine
	Vinorelbine
	*Belantamab mafodotin*
	*Dostarlimab*
	*Emapalumab*
	*Luspatercept*
	*Margetuximab*
	*Nivolumab/relatlimab*
	*Pertuzumab/trastuzumab and hyaluronidase*
	*Pixantrone*
	*Rituximab and hyaluronidase*
	*Siltuximab*
	*Trastuzumab and hyaluronidase*
	*Valrubicin*
	*Vincristine* (liposomal)

Agents in *italics* are not approved for clinical practice in Japan

FOLFIRINOX,  FOLFOXIRI: 5-fluorouracil, folinic acid, irinotecan, and oxaliplatin; S-1: tegafur, gimeracil, and oteracil

^*^Carboplatin (AUC ≥ 4) is in the high end of the moderate category for emetogenicity

^**^Sacituzumab govitecan and trastuzumab deruxtecan are in the high end of the moderate category for emetogenicity, and with the accumulation of future clinical trial results on antiemetic therapy, there is a possibility that they may be considered as candidates for the application of triple combination therapy including NK1RA

**Table 5:** Emetic risk category for oral antineoplastic agents

JSCO emetic risk category	Agent (Regimen)
High emetic risk (emetic frequency: 90% <)	Procarbazine
	*Hexamethylmelamine*
Moderate emetic risk (emetic frequency: 30% < –90%)	Bosutinib
	Busulfan(4 mg/day ≤)
	Ceritinib
	Crizotinib
	Cyclophosphamide
	Estramustine
	Imatinib
	Lenvatinib
	Mitotane
	Niraparib
	Olaparib
	Panobinostat
	Selumetinib
	Temozolomide
	Trifluridine・tipiracil (TAS-102)
	*Avapritinib*
	*Azacytidine*
	*Enasidenib*
	*Fedratinib*
	*Ivosidenib*
	*Lomustine*
	*Midostaurin*
	*Mobocertinib*
	*Rucaparib*
	*Selinexor*
	*Vinorelbine*
Low emetic risk (emetic frequency: 10–30%)	Abemaciclib
	Afatinib
	Alectinib
	Axitinib
	Binimetinib
	Busulfan (< 4 mg/day)
	Cabozantinib
	Capecitabine
	Capmatinib
	Dabrafenib
	Encorafenib
	Entrectinib
	Etoposide
	Everolimus
	Fludarabine
	Futibatinib
	Ibrutinib
	Ixazomib
	Lapatinib
	Lenalidomide
	Nilotinib
	Palbociclib
	Pazopanib
	Pemigatinib
	Ponatinib
	Quizartinib
	Regorafenib
	Sunitinib
	Tegafur・gimeracil・oteracil (S-1)
	Tegafur・uracil (UFT)
	Thalidomide
	Vandetanib
	Venetoclax
	Vorinostat
	*Alpelisib*
	*Cobimetinib*
	*Duvelisib*
	*Erdafitinib*
	*Glasdegib*
	*Idelalisib*
	*Neratinib*
	*Pacritinib*
	*Pexidartinib*
	*Ribociclib*
	*Ripretinib*
	*Sonidegib*
	*Talazoparib*
	*Tivozanib*
	*Topotecan*
	*Tucatinib*
Minimal emetic risk (emetic frequency: < 10%)	Acalabrutinib
	Asciminib
	Bexarotene
	Brigatinib
	Dacomitinib
	Dasatinib
	Erlotinib
	Forodesine
	Gefitinib
	Gilteritinib
	Hydroxycarbamide (hydroxyurea)
	Larotrectinib
	Lorlatinib
	Melphalan
	Mercaptopurine
	Methotrexate
	Osimertinib
	Pimitespib
	Pomalidomide
	Ruxolitinib
	Selpercatinib
	Sorafenib
	Sotorasib
	Tazemetostat
	Tepotinib
	Tirabrutinib
	Trametinib
	Tretinoin
	Tucidinostat
	Valemetostat
	Vemurafenib
	*6-Thioguanine*
	*Belzutifan*
	*Chlorambucil*
	*Decitabine and cedazuridine*
	*Vismodegib*
	*Zanubrutinib*

Agents in *italics* are not approved for clinical practice in Japan

### Background questions and future research questions with statements

BQs and FQs are shown in Table 6.

**Table 6:** Background questions and future research questions with statements

Question no.	Questions	Statements	Agreement rate	
			%	Number
BQ1	What antiemetic therapies are recommended for highly emetogenic risk antineoplastic agents?	For highly emetogenic risk antineoplastic agents, a four-drug combination therapy using olanzapine, a 5-HT3 receptor antagonist, an NK1 receptor antagonist, and dexamethasone is administered. In cases where the use of olanzapine is challenging, a three-drug combination therapy with a 5-HT3 receptor antagonist, an NK1 receptor antagonist, and dexamethasone is administered	100	24/24
BQ2	What factors should be considered when choosing 5-HT3 receptor antagonists for highly emetogenic risk antineoplastic agents?	For highly emetogenic risk antineoplastic agents, in the context of three-drug combination therapy, the acute antiemetic effect is nearly equivalent between first-generation (e.g., granisetron) and second-generation (e.g., palonosetron) agents. However, there is a tendency for palonosetron to exhibit a better delayed antiemetic effect. In cases of four-drug combination therapy, either first-generation or second-generation agents can be chosen. Yet, in situations where it is necessary to shorten the administration period of dexamethasone or when the use of olanzapine is challenging, palonosetron is prioritized	100	24/24
BQ3	What antiemetic therapies are recommended for moderately emetogenic risk antineoplastic agents?	For acute nausea and vomiting associated with moderate emetogenic risk antineoplastic agents, a combination of a 5-HT3 receptor antagonist and dexamethasone is administered. Carboplatin (AUC ≥ 4), which is at the high end of the moderate emetogenicity category, is administered as a triplet, with an NK1 receptor antagonist added to the regimen	100	24/24
BQ4	What factors should be considered when choosing 5-HT3 receptor antagonists for moderately emetogenic risk antineoplastic agents?	For moderately emetogenic risk antineoplastic agents, a two-drug combination therapy using the second-generation 5-HT3 receptor antagonist, palonosetron, and dexamethasone is administered. However, when adding an NK1 receptor antagonist, the first-generation 5-HT3 receptor antagonist can also be chosen	100	24/24
BQ5	What antiemetic therapies are recommended for low to minimal emetogenic risk antineoplastic agents?	There is no clear evidence for prophylactic antiemetic therapy for low emetogenic risk antineoplastic agents, but in actual clinical practice, antiemetics such as dexamethasone and 5-HT3 receptor antagonists are widely administered. For minimal emetogenic risk antineoplastic agents, prophylactic antiemetic therapy is not administered	100	25/25
BQ6	What antiemetic therapies are recommended for anticipatory nausea and vomiting?	Optimal management involves achieving complete control of acute and delayed nausea and vomiting caused by cancer chemotherapy, with the goal of preventing patients from experiencing these symptoms. In cases of anticipatory nausea and vomiting, benzodiazepine anxiolytics are administered	100	24/24
BQ7	What antiemetic therapies are recommended for nausea and vomiting induced by radiation therapy?	Perform an emetogenic risk classification based on the site of radiation exposure and implement antiemetic therapy tailored to the risk. For high risk (total body irradiation), it is recommended to proactively administer 5-HT3 receptor antagonists and dexamethasone for prevention. For moderate risk (e.g., upper abdomen, craniospinal irradiation), it is advisable to administer 5-HT3 receptor antagonists prophylactically, and concurrent use of dexamethasone may also be considered	100	24/24
BQ8	What factors should be considered when selecting the administration route for antiemetic agents?	There is no difference in the antiemetic effects of 5-HT3 receptor antagonists and NK1 receptor antagonists or in systemic side effects between intravenous and oral administration at approved routes and doses, and the choice of route should be based on the patient's condition	100	19/19
BQ9	What notable side effects should be considered for antiemetic agents?	Notable side effects of antiemetics include constipation and headache with 5-HT3 receptor antagonists and NK1 receptor antagonists, injection site reactions with fosaprepitant due to peripheral intravenous administration, somnolence and dizziness with olanzapine, insomnia and transient hyperglycemia with dexamethasone, and extrapyramidal symptoms (akathisia, acute dystonias, etc.) with metoclopramide	100	23/23
BQ10	How is antiemetic therapy conducted in chemothrapy involving the concomitant use of immune checkpoint inhibitors?	If immune checkpoint inhibitors are co-administered, antiemetic therapy should be selected based on the emetogenic risk of chemotherapy. Do not reduce the dose of dexamethasone antiemetic therapy because of the administration of immune checkpoint inhibitors	100	24/24
BQ11	What patient-related factors influence the effectiveness of antiemetic therapy?	Patient-related factors that may decrease the effectiveness of antiemetic therapy include young age, female gender, non-habitual alcohol consumption, and a history of motion sickness or morning sickness. Consider reinforcing antiemetic therapy tailored to the patient's background	100	22/22
BQ12	What support is required for controlling nausea and vomiting occurring outside the hospital, such as at home?	Support patients in appropriately assessing their symptoms and encourage them to promptly contact or visit the hospital in case of severe symptoms or concerns. Provide guidance on the proper use of rescue medications to help patients control nausea and vomiting even at home	100	19/19
BQ13	What information and support are needed to promote effective self-care by patients for nausea and vomiting?	In addition to explanations from the physician, the healthcare team, including nurses and pharmacists, should provide continuous information and support before the initiation of therapy. This involves detailing the expected severity, timing, and duration of nausea and vomiting; their impact on daily life; types of antiemetic agents and their side effects; emergency contact procedures; and lifestyle adjustments. While utilizing educational materials that patients can refer to as needed, tailor the approach based on individual needs	100	19/19
FQ1	Is the administration of 5-HT3 receptor antagonists recommended for the prevention of nausea and vomiting associated with low emetogenic risk antineoplastic agents?	For the prevention of nausea and vomiting associated with low emetogenic risk antineoplastic agents, there is no clear evidence, but in actual clinical practice, dexamethasone and 5-HT3 receptor antagonists are widely administered	100	22/22
FQ2	Is the administration of antiemetic agents recommended for the prevention of nausea and vomiting associated with oral anticancer drugs?	There is no evidence to support the use of antiemetics to prevent nausea and vomiting caused by oral antineoplastic agents. Management includes prescribing rescue medications and adjusting dosages or interrupting treatment as needed	100	22/22
FQ3	Is the additional administration of olanzapine recommended in cases where breakthrough nausea and vomiting occur despite the administration of olanzapine for the prevention of nausea and vomiting?	There is no evidence to recommend the addition of olanzapine after initial treatment with olanzapine for breakthrough nausea and vomiting. Consider administration of antiemetics other than olanzapine for management	100	22/22

### Clinical questions and recommendations

### CQ1: Is the addition or concurrent use of olanzapine recommended for the prevention of nausea and vomiting associated with highly emetogenic risk antineoplastic agents using a triplet antiemetic regimen (a 5-HT3 receptor antagonist + an NK1 receptor antagonist + dexamethasone)?

Recommendation: We recommend the addition or concurrent use of olanzapine to a triplet antiemetic regimen to prevent nausea and vomiting associated with antineoplastic agents with high emetogenic risk.

[Strength of recommendation: 1; Quality of evidence: B; Agreement rate: 95.7% (22/23)].

### CQ2: Is it recommended to shorten the administration duration of dexamethasone to one day for the prevention of nausea and vomiting associated with highly emetogenic risk antineoplastic agents?

Recommendation: We suggest shortening the duration of dexamethasone administration to one day to prevent the nausea and vomiting associated with antineoplastic agents with a high emetogenic risk, especially in the case of AC regimens.

[Strength of recommendation: 2; Quality of evidence: B, Agreement rate: 95.5% (21/22)].

### CQ3: Is the administration of an NK1 receptor antagonist recommended for the prevention of nausea and vomiting associated with moderately emetogenic risk antineoplastic agents?

Recommendation: We recommend the administration of NK1 receptor antagonists to prevent the nausea and vomiting associated with carboplatin regimens in moderate-emetogenic-risk antineoplastic agents.

[Strength of recommendation: 1; quality of evidence: A, Agreement rate: 100% (22/22)].

### CQ4: Is the addition or concurrent use of olanzapine to the triplet antiemetic regimen (a 5-HT3 receptor antagonist + an NK1 receptor antagonist + dexamethasone) recommended for the prevention of nausea and vomiting associated with moderately emetogenic risk antineoplastic agents?

Recommendation: We suggest the addition or concurrent use of olanzapine to the triplet antiemetic regimen to prevent the nausea and vomiting associated with moderate-risk emetogenic antineoplastic agents.

[Strength of recommendation: 2; Quality of evidence: C, Agreement rate: 87.5% (21/24)].

### CQ5: Is the addition or concurrent use of olanzapine to the doublet antiemetic regimen (a 5-HT3 receptor antagonist + dexamethasone) recommended for the prevention of nausea and vomiting associated with moderately emetogenic risk antineoplastic agents?

Recommendation: No consensus was reached.

[Strength of recommendation: not granted; quality of evidence: C, Agreement rate: N/A (Two votes were taken. No consensus was reached.)].

### CQ6: Is it recommended to shorten the administration duration of dexamethasone to one day for the prevention of nausea and vomiting associated with moderate emetogenic risk antineoplastic agents?

Recommendation: We recommend shortening the duration of dexamethasone administration to one day to prevent the nausea and vomiting associated with moderate-risk emetogenic antineoplastic agents, especially when administering palonosetron as a 5-HT3 receptor antagonist.

[Strength of recommendation: 1; Quality of evidence: B, Agreement rate: 90.5% (19/21)].

### CQ7: Is it recommended to omit the administration of an NK1 receptor antagonist for the prevention of nausea and vomiting in R ± CHOP regimens?

Recommendation: We suggest not to omit the administration of an NK1 receptor antagonist for the prevention of nausea and vomiting in R ± CHOP regimens.

[Strength of recommendation: 2; Quality of evidence: C; Agreement rate: 91.7% (22/24)].

### CQ8: Is the administration of metoclopramide recommended for breakthrough nausea and vomiting?

Recommendation: We suggest the administration of metoclopramide for breakthrough nausea and vomiting.

[Strength of recommendation: 2; Quality of evidence: B, Agreement rate: 95.8% (23/24)].

### CQ9: Is daily antiemetic therapy recommended for patients receiving daily intravenous administrations of cytotoxic antineoplastic agents?

Recommendation: We recommend the implementation of daily antiemetic therapy in patients receiving daily intravenous cytotoxic antineoplastic agents.

[Strength of recommendation: 1; Quality of evidence: D, Agreement rate: 95.8% (23/24)].

### CQ10: Is the concurrent use of non-pharmacological therapy recommended for the prevention of nausea and vomiting?

Recommendation: We suggest not to perform non-pharmacological interventions for the management of nausea and vomiting.

[Strength of recommendation: 2; Quality of evidence: D, Agreement rate: 83.3% (20/24)].

### CQ11: Is non-pharmacological therapy recommended for anticipatory nausea and vomiting?

Recommendation: We suggest not to perform non-pharmacological interventions for anticipatory nausea and vomiting.

[Strength of recommendation: 2; Quality of evidence: D, Agreement rate: 95.8% (23/24)].

### CQ12: Is the use of patient-reported outcomes recommended for the assessment of nausea and vomiting?

Recommendation: We recommend using patient-reported outcomes to assess nausea and vomiting.

[Strength of recommendation: 1; quality of evidence: B, Agreement rate: 100% (22/22)].

## Summary

**Fig. 1:**
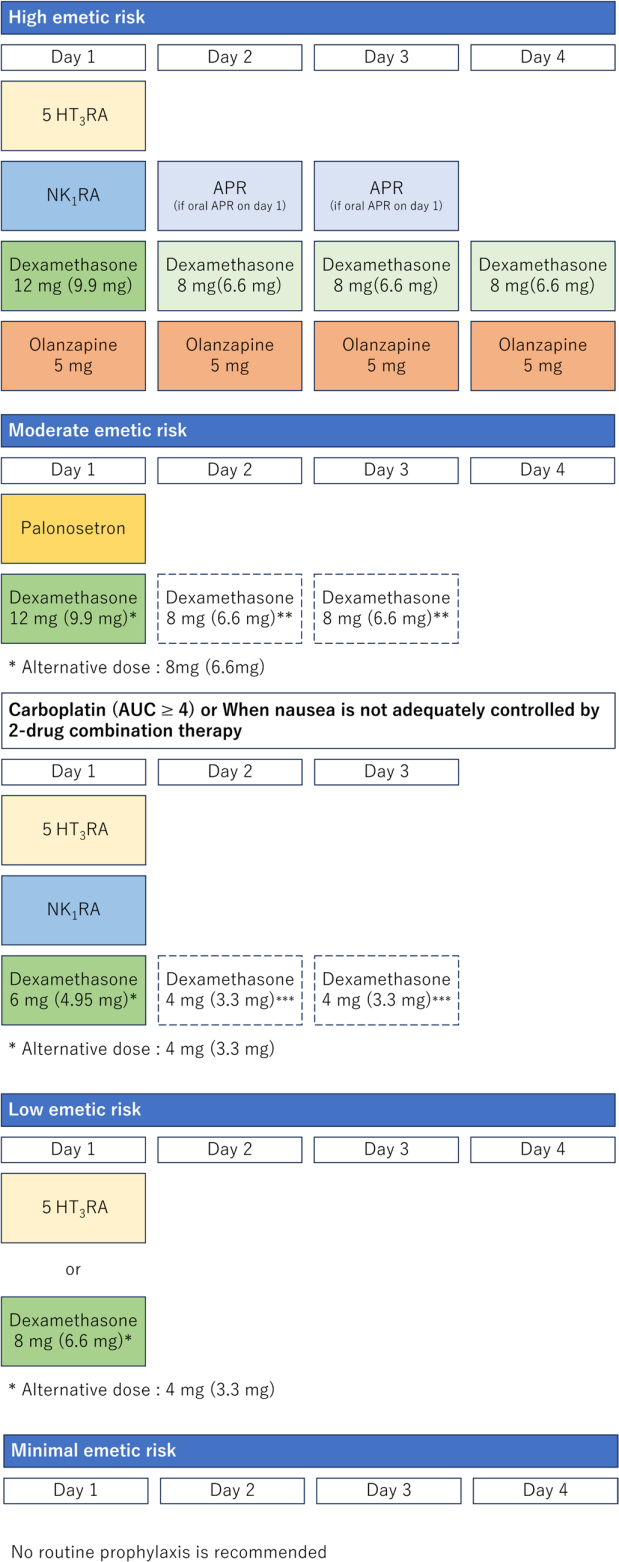
Schematic diagram of antiemetic treatments for intravenous antineoplastic agents. * Alternative dexamethasone dose. ** If first generation 5HT3RA is administered. *** Optional dose of dexamethasone. The diagrams show standard examples of antiemetic treatment regimens. Flexible modifications are necessary, depending on the specific condition of each patient. The recommended dose of dexamethasone has been specified for oral (intravenous) administration. Intravenous dexamethasone includes 3.3 mg/mL of dexamethasone out of a total of 4 mg/mL of dexamethasone sodium phosphate

Adult antiemetic dosing information is listed in Table 7, and the standard model for antiemetic treatment regimens is detailed in the four diagrams shown in Fig. 1.

**Table 7:** Antiemetic dosing for adults

Classification	Agents	Dose on day of chemotherapy	Dose on subsequent days
5HT_3_ receptor antagonist	Ondansetron	4 mg IV or oral	
	Granisetron	40 μg/kg IV or 2 mg oral	
	Ramosetron	0.3 mg IV or 0.1 mg oral	
	Palonosetron	0.75 mg IV	
NK_1_ receptor antagonist	Aprepitant	125 mg oral	80 mg oral on days 2–3
	Fosaprepitant	150 mg IV	
	Fosnetupitant	235 mg IV

## Discussion

This manuscript presents an English summary of the Japan Society of Clinical Oncology Clinical Practice Guidelines for Antiemesis 2023.

In the present guidelines, the emetic risk classification has been revised to incorporate new antineoplastic agents and chemotherapy regimens. Currently, classification is based on the emetic risk during the acute phase without antiemetic therapy. However, obtaining such data during clinical trials for the development of antineoplastic agents is challenging.

It is important to note that sacituzumab govitecan and trastuzumab deruxtecan are at the high end of the moderate category for emetogenicity, and with the accumulation of future clinical trial results on antiemetic therapy, there is a possibility that they may be considered as candidates for the application of triple combination therapy including NK1 receptor antagonists. Currently, these agents are classified as having moderate emetic risk due to insufficient evidence regarding their emetogenic potential. However, contingent on the results of future clinical trials, they may be reclassified into the high emetic risk category. Therefore, when using these agents, it is crucial to carefully monitor the patient's condition and flexibly adjust the antiemetic therapy as needed, such as considering the concomitant use of NK1 receptor antagonists. As new evidence on antiemetic therapy accumulates, the content of the guidelines will need to be updated accordingly. Readers are advised to stay informed about the latest findings regarding emetogenicity.

The emetogenic properties of certain combination chemotherapy are classified based on the incidence and severity of emesis observed with antiemetic therapy commonly used in clinical trials and in real-world clinical practice.

We had initially proposed 15 CQs for this update. However, because insufficient evidence prevented the completion of a systematic review for three of them, the three were categorized as FQs. Urgent attention is warranted for conducting clinical trials to address all three questions.

Prevention of nausea and vomiting in patients undergoing cancer chemotherapy is critical not only for treatment efficacy, but also for maintaining the overall quality of life. These guidelines are intended to promote and facilitate appropriate antiemetic therapy in clinical practice and serve as a supportive resource for clinicians and medical staff to make compassionate decisions tailored to individual patients undergoing cancer chemotherapy. By promoting the implementation of effective strategies, we hope to contribute to the overall success of cancer treatment and enhance the well-being of the patients undergoing this challenging therapeutic journey.

## References

[1] Takeuchi H, Saeki T, Aiba K (2016). Japanese Society of Clinical Oncology Clinical practice guidelines 2010 for antiemesis in oncology: Executive summary. Int J Clin Oncol.

[2] Aogi K, Takeuchi H, Saeki T (2021). Optimizing antiemetic treatment for chemotherapy-induced nausea and vomiting in Japan: Update summary of the 2015 Japan Society of Clinical Oncology Clinical Practice Guidelines for Antiemesis. Int J Clin Oncol.

[3] Minds Clinical Practice Guideline Development Manual 2017. https://minds.jcqhc.or.jp/ (Japanese) [accessed April 1, 2024]

[4] Japan Society of Clinical Oncology Clinical practice guidelines for antiemesis. http://www.jsco-cpg.jp/item/29/index.html (Japanese) [accessed April 1, 2024]

[5] Jaeschke R, Guyatt GH, Dellinger P (2008). Use of GRADE grid to reach decisions on clinical practice guidelines when consensus is elusive. BMJ.

